# Reconstructive Surgery in a Patient with Persistent Cloaca

**Published:** 2011-11-27

**Authors:** Altaf Begum, Afzal Sheikh, Bilal Mirza

**Affiliations:** Department of Pediatric Surgery, The Children's Hospital and the Institute of Child Health Lahore, Pakistan

**Keywords:** Persistent cloaca, Urogenital sinus, Anorectal malformation, Fecal continence

## Abstract

Cloacal malformations are challenging as to the surgical correction. A case of cloacal malformation who underwent reconstructive surgery is being reported. The patient had colostomy in the neonatal period and reconstruction was performed at the age of 6 year. The surgical management included abdomino-perineal anorecto-urethro-colo-vaginoplasty. The patient is fully continent of urine and achieved fair continence of feces at 9 months of follow up.

## INTRODUCTION

Persistent cloaca is an uncommon malformation with a wide spectrum of urogenital and hind gut anomalies. It is placed under the heading of complex/rare malformations in the Krickenbeck’s classification of anorectal malformations [1]. The clinical presentation is of imperforate anus with a single perineal opening through which urine and meconium are passed. Unlike other anorectal malformations, surgical treatment is demanding as urinary and fecal incontinence are frequently reported following operation. The ultimate goal of treatment includes achieving satisfactory bowel and urinary control as well as normal sexual functions at maturity [1, 2, 3]. In this manuscript the management of the index anomaly is reported who achieved normal urinary and fair fecal continence.

## CASE REPORT

A six-year-old female presented for definitive procedure of persistent cloaca (Fig. 1). She had colostomy on 5th day of life. Ultrasound of abdomen was reported as normal. Distal colostogram showed high recto-cloacal fistula (Fig. 2). Endoscopic evaluation revealed an opening at bladder neck. At operation anal sphincter was identified by nerve stimulation and dissection started by making anterior sagittal incision. The sites for the future vagina and ano-rectum were made. Abdomen was then opened by mobilizing the stoma. Urinary bladder was opened and ureteric catheterization done to avoid their damage during surgery. The anomaly was identified as high confluence of rectum and vagina opening into the cloaca at the level of bladder neck (Fig. 3). The opening of vagina was very minute that could not be identified on endoscopy as well as at operation. Distal loop of the colon was mobilized and detached from common cloaca. It was tailored distally, to be used as vaginal substitute and pulled down at the perineum, while its proximal end anastomosed with lower end of the vagina that was about 1/3rd in length. Similarly proximal loop of colon mobilized to bring it down as ano-rectum, through the already identified site for anus. Feminizing clitoroplasty was added. The common channel was left as urethra. Finally perineal body was constructed (Fig. 4). Patient had uneventful recovery.

**Figure:**
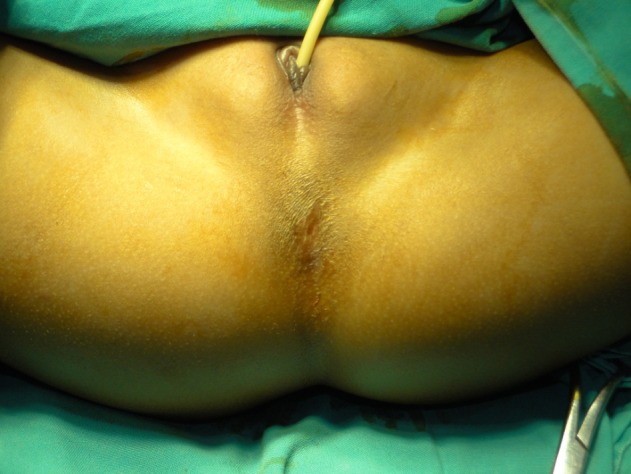
Figure 1: Preoperative figure showing single perineal opening.

**Figure:**
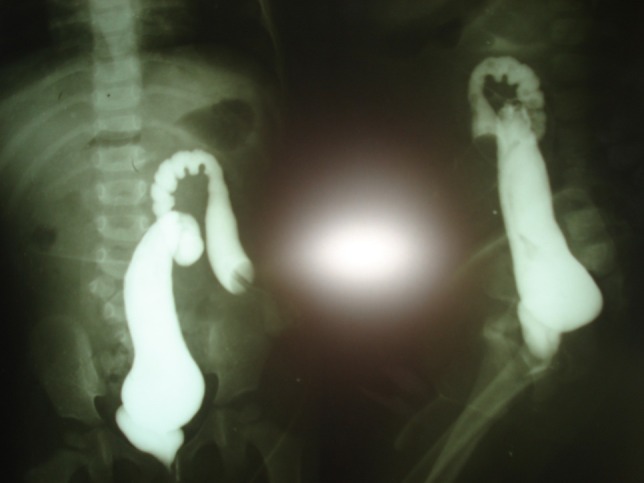
Figure 2: Distal loopogram showing high insertion of rectum into common channel.

**Figure:**
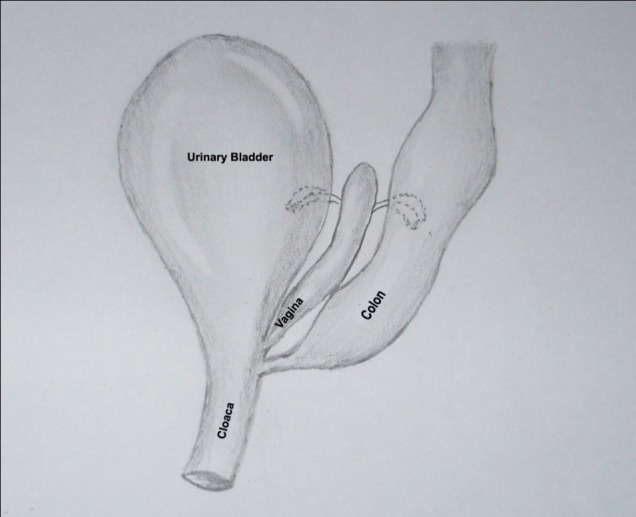
Figure 3: Illustration of the cloacal malformation.

**Figure:**
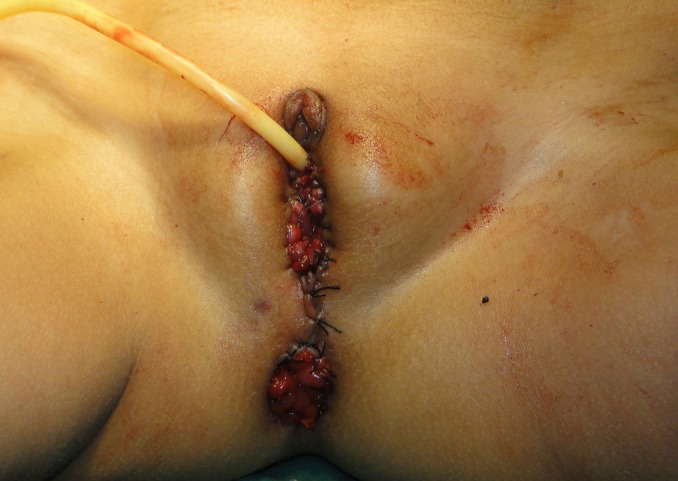
Figure 4: Postoperative showing anorecto-colovagino-urethroplasty.

Examination done after 02 weeks of surgery showed healed patent vaginal and anal passages. On 10th week post operative visit, the mother was satisfied the urinary continence. She observed occasional soiling (Kelly’s continence score 3). She was on follow up and on vaginal dilatation program.

## DISCUSSION

Common cloaca or persistent cloaca is rare congenital malformation characterized by confluence of rectum, vagina and the distal urinary tract into a single common channel. Cloacal anomalies occur in 1:250,000 live births [1]. Management of these defects is a challenge to pediatric surgeons. The definitive treatment in most centers is a single stage reconstruction though some surgeons opt for repairing the anorectal anomaly initially and leave urogenital sinus repair for later date. The surgical management is planned after carefully identifying the anatomy specially measuring length of common channel, level of insertions of urinary channel, vagina and rectum, and associated urogenital anomalies. The patients with cloacal malformations are categorized in two groups depending upon the length of the common channel. Those having common channel shorter than 3cm (more than 60% of entire group) can be repaired by posterior sagittal approach. The second group has a length of the common channel more than 3cm where the total urogenital mobilization from perineum will not be enough to repair the malformation, so the common channel is left intact to be used as urethra, and colovaginoplasty along with anorectoplasty is performed as done in our case [3, 4, 5].

Fecal and urinary incontinence is a major problem in high variety anorectal malformations. Their intensity is even higher in cases of persistent cloaca. Almost 60% patients have incontinence of variable degrees. The incidence is usually higher in cases of higher confluence where an abdomino-perineal approach is used [1, 5]. In our case despite extensive pelvic and perineal dissection the patient achieved fair bowel and normal urinary control. This may be attributed to the well developed perineal muscles and sparing of the urethral sphincter along with good surgical repair. 

## Footnotes

**Source of Support:** Nil

**Conflict of Interest:** None declared

## References

[1] ( 2001). Buhilla P, Torres PC, Bruned BJ, Emparan G, DeSalazar C, Castro LC. Total mobilization of the urogenital sinus in the treatment of cloaca. An Esp Pediatr.

[2] ( 1977). Kay R, Tank ES. Principles of management of persistent cloaca in the female newborn. J Urol.

[3] ( 2005). Masuko T, Higashimoto Y, Iwai J. Single-stage operation without temporary colostomy for persistent cloaca with short common channel. Pediatr Surg Int.

[4] ( 2007). Malik A, Iqbal Z, Hameed S, Khalid C, Iftikhar A, Hussain M. Persistent cloaca with complete duplex ectopic ureters: A rare presentation. J Med Rehabilit.

[5] ( 2005). Levitt MA, Pena A. Pitfalls in the management of newborn cloacas. Pediatr Surg Int.

